# High Reproducibility and Agreement of Meal Duration, Number of Chews, and Chewing Tempo Measured with a Standardized Test Meal

**DOI:** 10.3390/nu17152438

**Published:** 2025-07-25

**Authors:** Kanako Deguchi, Kenichiro Ikeda, Megumi Aoshima, Eri Hiraiwa, Chisato Ono, Chihiro Ushiroda, Risako Yamamoto-Wada, Katsumi Iizuka

**Affiliations:** 1 Department of Clinical Nutrition, Fujita Health University, Toyoake 470-1192, Japan; kanasakuran@gmail.com (K.D.); 51022005@fujita-hu.ac.jp (K.I.); 51021001@fujita-hu.ac.jp (M.A.); 51022099@fujita-hu.ac.jp (E.H.); chihiro.ushiroda@fujita-hu.ac.jp (C.U.); risako.wada@fujita-hu.ac.jp (R.Y.-W.); 2 Faculty of Medicine, Fujita Health University, Toyoake 470-1192, Japan; 3 Department of Medical Technology, Fujita Health University Haneda Clinic, Tokyo 144-0041, Japan; chisato.ono@fujita-hu.ac.jp; 4 Food and Nutrition Service Department, Fujita Health University Hospital, Toyoake 470-1192, Japan

**Keywords:** food test, meal duration, chewing tempo, number of chews, number of bites, reproducibility, agreement

## Abstract

Background/Aim: To date, there have been no data regarding the reproducibility or agreement of meal duration when a test meal is eaten. To confirm the reproducibility and agreement of the meal duration, number of chews, chewing tempo, and number of bites of a test meal, we performed a prospective observation study. Methods: We measured the duration, number of chews, chewing tempo, and number of bites of a test meal (salmon bento) among 33 participants (male: 15; female: 18) aged 20–60 years who ate twice at 2-week intervals to verify the agreement (by Bland-Altman (BA) analysis) and reproducibility (intraclass correlation coefficient (ICC)) by sex. Results: The meal duration (s) and number of bites (times) were significantly greater in the female group (560.4 (128.7) and 731.9 (266.3), *p* = 0.023; 17.1 (9.9) vs. 26.4 (13.7), *p* = 0.036), and the number of chews tended to be greater in the female group (752.5 (203.3) vs. 938.1 (375.9), *p* = 0.083). Meal duration was positively associated with the number of chews (0.64 [0.53, 0.74], *p* < 0.001) and bites (10.4 [5.1, 15.8], *p* < 0.001). For both sexes, the % error calculated via BA analysis was high for meal duration, the number of chews, and the chewing tempo (21.4 and 13.4%; 16.5 and 18.5%; and 6.8 and 5.3%, respectively) and low for the number of bites (37.9 and 68.9%). The ICCs were high for meal duration (0.73 and 0.90), the number of chews (0.76 and 0.89), and the chewing tempo (0.76 and 0.90), and low for the number of bites (0.84 and 0.69). Moreover, systematic and proportional errors were found only for the number of bites in the female group (median_difference with 95% CI: −9.00 (−13.00, −2.00); −0.320 (−0.45, −0.093)). Conclusions: Although the sample size was small due to the exploratory nature of the study, meal duration, number of chews, and chewing tempo had high reproducibility and agreement, at least when this test meal was consumed. These measures may indicate individual-specific eating behavior.

## 1. Introduction

Diet, exercise, and behavioral therapies are important in the treatment of obesity [1]. Dietary treatment for obesity includes eating three regular meals a day, eating a nutritionally balanced diet, consuming vegetables and seaweed, avoiding high-calorie beverages and snacks, and avoiding alcohol [1]. In addition, the act of chewing food well and eating slowly is known to suppress hunger and increase satisfaction, in addition to reducing energy intake [2,3]. Compared with normal-weight individuals, obese individuals tend to chew less frequently and eat faster, possibly due to impaired satiety feedback [4]. Chewing activates oropharyngeal and neural signals involved in satiety and digestion, potentially influencing hypothalamic control of hunger [5]. Moreover, slow eating leads to decreases in body mass index (BMI) and postprandial glucose spikes and promotes insulin resistance in healthy individuals [6,7]. Prolonged chewing improves satiety by increasing gut hormone release (e.g., GLP-1, PYY) and reducing ghrelin levels [8].

With respect to Japanese dining etiquette, people are instructed to put their chopsticks on the chopstick rest when they have food in their mouths and not to pick up the next piece of food [9]. Moreover, in Japan, eating is traditionally a mindful activity, and chewing thoroughly is encouraged because many traditional Japanese foods, such as dried fish, brown rice, and root vegetables, are high in texture and require effort to chew. In Japan, the abovementioned techniques are often included in nutritional guidance for obese patients. In fact, it has been reported that energy intake decreases as meal duration increases [10]. Furthermore, in our study, we found that the number of chews and meal duration were positively related [11,12]. Therefore, meal duration, the number of chews, and chewing tempo are recognized as factors that reflect eating behavior as well as energy intake.

Although the meal duration and number of chews for a given test meal may be useful indicators of eating behavior, the reproducibility and consistency of this test are unknown since each test is conducted only once. With respect to the reproducibility of dietary tests, several studies have examined the reproducibility of dietary energy intake, and high reproducibility and agreement have been reported [13,14,15,16]. Therefore, it is necessary to verify the reproducibility and agreement of the meal duration and number of chews when a test meal is used.

The differences in eating behavior between the sexes are also important [17,18,19,20,21,22]. Compared with men, women tend to eat more slowly, chew more, and take more time to eat [17,18]. In our previous study, women tended to take longer to eat, chew more during a meal, and eat more often than men did, but there was no difference in chewing tempo between men and women, indicating that there are differences in these parameters [11,12]. In terms of food preferences, males tend to prefer meat and high-fat foods, whereas females tend to prefer vegetables, fruits, and sweet foods [19,20,21,22]. In women, sensitivity to the food environment is also influenced by emotions and stress [23]. For example, it is known that women feel more satisfied after eating, that their cognitive control areas, such as the prefrontal cortex, respond strongly, that they are visually sensitive to the eating environment, and that correlations with the amount of food consumed have also been reported [23]. Therefore, sex differences may also influence the reproducibility of the diet test.

The aim of this study was to assess the reproducibility and agreement of the meal duration, number of chews, chewing tempo, and number of bites during a food test for each sex. The participants were asked to consume the test meal (salmon bento box lunch) twice, with a two-week interval. We estimated reproducibility and consistency via Bland-Altman analysis and the intraclass correlation coefficient (ICC). If we can prove the reproducibility and consistency of the eating behavior metrics when consuming the test food, the eating behavior metrics appear to be viable parameters for representing individual-specific eating behavior.

## 2. Materials and Methods

### 2.1. Study Design and Setting

This was a prospective observational study that aimed to evaluate the reproducibility of repeated measurements of meal-related behaviors. The measurements were conducted in a controlled laboratory setting over two separate sessions across 14 days.

### 2.2. Participants

This study was a prospective intervention trial in which 33 participants aged 20–60 years (male: *n* = 15; female: *n* = 18) were recruited from the faculty and staff of Fujita Health University. The study was conducted from 6 February 2025 to 22 May 2025. We recruited individuals who actually work at the hospital to use the results of this study to guide our employees’ future health. Consequently, we applied the following criteria. The inclusion criteria were as follows: 1. Individuals (employees and students) aged 20–65 years at the time of consent who provided written consent to participate after being informed and demonstrating comprehension. Individuals who were deemed unsuitable for participation in this study by a physician were excluded. We confirmed verbally that individuals with food allergies or severe diabetes or kidney disease were not eligible to participate in the study. Considering the very limited number of staff over 65 years of age and the effect of age, individuals older than 65 years were excluded from this study. Although not included in the exclusion criteria, perhaps owing to the small number of participants, individuals with anorexia nervosa, bulimia nervosa, or preexisting diabetes or kidney disease were not included. The study was conducted according to the principles of the Declaration of Helsinki and was approved by the Research Ethics Committee of Fujita Health University (approval number; HM24-458, approval date: 28 January 2025). All patients provided written informed consent before inclusion in the study. This study was registered with the UMIN Clinical Trial Registry (UMIN000056626, registered on 7 February 2025).

### 2.3. Variables

The following four meal-related behavioral indicators were assessed:

1. Meal duration (total time spent eating) (s)
2. Number of chews (total number of chews) (time)
3. Chewing tempo (number of chews per unit minutes) (cpm)
4. Number of bites (total number of bites) (times)

Each indicator was measured twice for each participant. Sex (male/female) was recorded and used for stratified analysis.

### 2.4. Measurement of Meal Duration, Numbers of Chews and Bites, and Chewing Tempo

As in our previous studies [8,9], in this study, a bitescan^TM^ device (Sharp Inc., Sakai, Osaka, Japan) was used to assess the meal duration, number of chews, number of bites, and chewing tempo in accordance with a previously established methodology [24,25]. The meal duration was recorded via a stopwatch and verified via synchronized video recording. Chewing metrics were measured with the Bitescan™ device (Sharp Inc., Sakai, Osaka, Japan) via validated algorithms [24,25]. The chewing tempo was defined as the number of chews per minute. The number of bites was defined as the number of times a person took a break from chewing during meals.

### 2.5. Brief-Type Self-Administered Diet History Questionnaire (BDHQ)

The brief-type self-administered diet history questionnaire (BDHQ) is a self-administered diet history questionnaire that is used in Japan to estimate baseline dietary patterns and control for habitual intake [26,27].

### 2.6. Experiments

**Figure 1:**
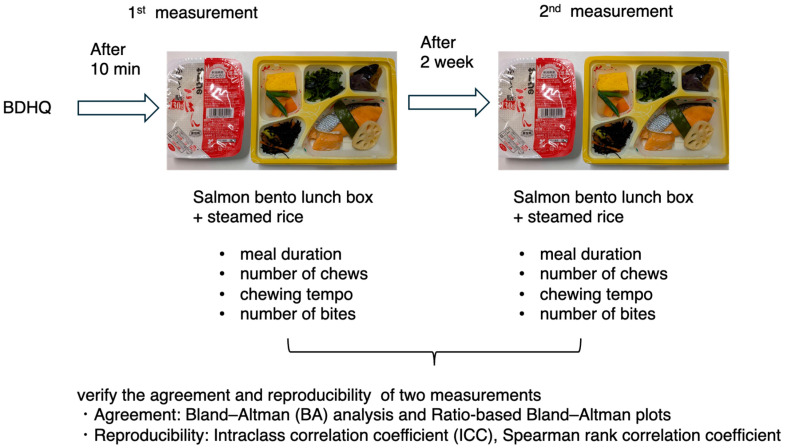
Procedure of this study. We measured the duration, number of chews, chewing tempo, and number of bites of a test meal among 33 participants aged 20–60 years (mean 41.6) who ate twice at 2-week intervals to verify the agreement and reproducibility in males (*n* = 15) and females (*n* = 18). BDHQ was measured, and the test meal measurement was conducted 10 min later. Two weeks later, the second test meal measurement was conducted. Measurement via the test meal was carried out after confirming that at least four hours had passed since breakfast.

Body weight was measured, the BDHQ was administered after ensuring that at least 4 h had passed since the participants had eaten breakfast, and the weight and handgrip strength of the participants were measured. Ten minutes later, the meal measurement experiment began (Figure 1). The participants first ate sake bento lunch boxes (nichirei, Tokyo, Japan; energy, 262 kcal; protein, 19.6 g; fat, 16.3 g; carbohydrate, 11.2 g) and steamed rice (sato no gohan) 130 g (Sato syokuhin, Niigata, Japan; energy, 191 kcal; protein, 2.7 g; fat, 0 g; carbohydrate, 44.1 g). The meal duration, number of chews, average chewing tempo, and number of bites were measured. After 2 weeks, the participants were instructed to repeat the experimental meal (Figure 1).

### 2.7. Data Sources and Measurement

Meal behaviors were recorded via video analysis, wearable sensors, and specific methods and were quantified according to standardized protocols. All the measurements were conducted under the same experimental conditions and instructions to minimize intersession variability.

### 2.8. Bias

To minimize measurement bias, the same instruments and procedures were used across both sessions. Analysts were blinded to the session order.

### 2.9. Study Size

A total of 33 participants were included in the analysis. No formal sample size calculation was performed, as this was an exploratory reproducibility analysis.

### 2.10. Statistics

Since the test meals in this study are different from those in previous papers, a multivariate analysis was conducted to first confirm whether meal duration, number of chews, number of bites, age, and gender are also related to the test meals used in this study. As there were only 33 study participants and only two variables could be examined at a time, the following three models were used for analysis. Specifically, multivariate linear regression analysis was performed with meal duration as the dependent variable and the number of chews (Model 1), mean chewing tempo (Model 2), number of bites (Model 3), and age (Model 4) as the independent variables adjusted for sex. IBM SPSS software version 29.0.2 (IBM, Armonk, NY, USA) was used for the statistical analysis.

When comparing two sets of data, checking the reproducibility is not enough; it is necessary to check the agreement by performing Bland-Altman analysis. A Bland–Altman plot (difference plot) in analytical chemistry or biomedicine is a method of data plotting used to analyze the agreement between two different assays. A Bland-Altman plot is identical to a Tukey mean-difference plot [28]. A nonparametric Bland-Altman analysis is a variation of the Bland-Altman method used to assess agreement between two measurement methods when the assumption of normality for the differences between measurements is not met. Nonparametric methods such as quantiles are used to define limits of agreement instead of relying on the mean and standard deviation [29].

To evaluate the reproducibility and agreement of the eating behavior indices (meal duration, number of chews, chewing tempo, and number of bites), Bland–Altman analysis was conducted using both absolute differences and ratio-based differences (differences divided by the means of the two measurements). Ratio-based Bland–Altman plots were used to account for interindividual scaling effects.

In addition to Bland–Altman analysis, the Wilcoxon signed-rank test was performed to assess systematic bias. The Spearman rank correlation coefficient and the intraclass correlation coefficient (ICC) were calculated as indicators of reproducibility. The ICC was computed via a two-way random effects model (agreement, single measurement), and its 95% CI was estimated by bootstrapping (2000 replicates). All analyses were performed for the total sample and stratified by sex.

### 2.11. Software

All analyses were conducted via R software (version 4.5.0 (11 April 2025); The R Foundation for Statistical Computing, Vienna, Austria) and RStudio (version 2025.05.0+496) using the following packages:

- dplyr v1.1.4 for data wrangling;
- boot v1.3-28.1 for bootstrap confidence intervals;
- irr v0.84.1 for ICC computation;
- ggplot2 v3.5.1 for plotting Bland–Altman plots;
- stats (base R) for the Wilcoxon and Spearman tests.

## 3. Results

### 3.1. Background Characteristics of the Participants

First, the background characteristics of the thirty-three participants (male: *n* = 15; female: *n* = 18) included in this study are as follows. The ages [mean (SD)] of the total, male, and female groups were 41.6 (8.4), 42.0 (8.2), and 41.2 (8.9) years, respectively. The BMIs [mean (SD)] of the total, male, and female groups were 22.5 (3.0), 23.7 (3.9), and 21.5 (2.7), respectively. The average estimated glomerular filtration rate (eGFR) and glycosylated hemoglobin (HbA1c), uric acid (UA), total cholesterol (TC), triglyceride (TG), and high-density lipoprotein cholesterol (HDL-C) levels were almost within normal ranges (eGFR: 83.1 (13.1) mL/min/1.73 m^2^, HbA1c: 5.4 (0.3)%, UA: 4.8 (1.4) mg/dL, T-Chol: 203.5 (31.1) mg/dL, TG: 103.8 (71.5) mg/dL, HDL-C: 64.3 (14.1) mg/dL) (Table 1). In terms of food consumption, the intakes of energy (kcal), protein (g), fat (g), carbohydrates (g), dietary fiber (g), and salt (g) were 1568.6 (432.0), 59.0 (17.0), 50.7 (13.3), 200.6 (65.3), 9.4 (3.4), and 9.1 (2.4), respectively (Table 1). Thus, most clinical indicators for participants were within normal ranges, suggesting that they were generally healthy.

Next, we determined the meal duration, number of chews, chewing tempo, and number of bites. For the paired samples *t* test, the 1st and 2nd meal durations (s) were 654.0 (229.0) and 696.3 (275.0), respectively. The 1st and 2nd numbers of chews (times) were similar (854.7 (319.3) and 880.4 (329.1), *p* = 0.38). The 1st and 2nd chewing tempos (cpm) were 82.6 (11.0) and 81.7 (12.1), respectively. The 1st and 2nd numbers of bites (times) were 22.2 (12.8) and 27.2 (22.3), respectively (Table 2).

### 3.2. Multivariate Analysis of Meal Duration and Other Factors (Number of Chews, Chewing Tempo, Number of Bites, Age, and Sex)

We previously reported that factors influencing diet were associated with sex, the number of chews, and the number of bites [11,12]. We reported the results of experiments conducted with pizza and hamburger bento [11,12], but since we used salmon bento in this study, we also examined the relationships between meal duration and the number of chews, chewing tempo, number of bites, age, and sex. Therefore, we conducted a multivariate analysis of the factors affecting meal duration. In Model 1, meal duration was associated with the number of chews (number of chews: 0.64 [0.53, 0.74], *p* < 0.001; sex: 53.8 [–12.48, 119.99]) (Table 3). In Model 2, meal duration was not related to chewing tempo but was associated with sex (182.6 [27.91, 337.2], *p* = 0.02) (Table 3). In Model 3, meal duration was associated with the number of bites (10.4 [5.1, 15.8], *p* < 0.001) (Table 3). In Model 4, meal duration was associated with age and sex (−9.2 [−17.9, −0.5], *p* = 0.04, 164.4 [18.8, 310.0], *p* = 0.03) (Table 3). Thus, consistent with our previous studies, in this study, the meal duration for salmon bento lunch boxes was associated with the number of chews, number of bites, sex, and age.

### 3.3. Bland–Altman Analysis

Next, we tested the reproducibility of the meal duration, number of chews, chewing tempo, and number of bites when the same food was eaten, which was the objective of the present study. In other words, we used Bland-Altman analysis to verify the validity of the second test and the first test. Ratio-based Bland-Altman analysis was also conducted to account for the wide range of measurement values.

In this study, the reproducibility and agreement of the meal duration, chewing count, eating tempo, and bite count were evaluated via Bland-Altman analysis and the Wilcoxon signed-rank test, and differences were compared between the male and female groups. Additionally, reproducibility was assessed via the ICC and Spearman’s rho, and both % error and the median ratio were calculated.

#### 3.3.1. Meal Duration

**Figure 2:**
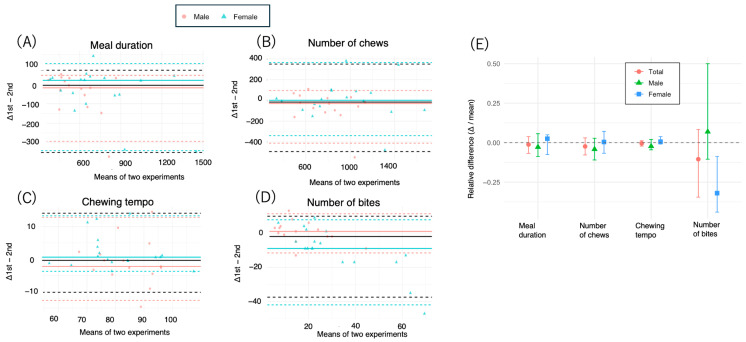
Bland-Altman plot showing the agreement between 1st and 2nd measurements. (**A**–**D**) We measured the duration, number of chews, chewing tempo, and number of bites of a test meal (salmon bento lunch box and steamed rice) among 33 participants aged 20–60 years (mean 41.6) who ate twice at 2-week intervals. The horizontal axis represents the mean of the two measurements, and the vertical axis shows their difference (measurements 1–2). The solid horizontal line indicates the median difference. The dashed lines represent the 2.5th and 97.5th percentiles (nonparametric limits of agreement). Red and blue indicate males and females, respectively. Δ1st–2nd: difference between the 1st and 2nd experiments. Values close to zero on the vertical axis indicate low proportional error, suggesting good agreement across the range of measurements. Black lines: overall (total) median (solid) and LOA (dashed); blue/red lines: sex-specific medians and LOA (with legend); data points: colored and shaped by sex. (**E**) The *x*-axis indicates the mean of the two values, and the *y*-axis shows the ratio of their difference to the mean (i.e., (measurement 1st–measurement 2nd)/mean), reflecting the proportional error. The error bars indicate the 95% confidence intervals estimated by bootstrapping (*n* = 2000 replicates). Bland–Altman ratio analysis was conducted via ratio-based differences (differences divided by the means of the two measurements). Ratio-based Bland–Altman plots were used to account for interindividual scaling effects. Δ: difference between the 1st and 2nd experiments. Values close to zero on the vertical axis indicate low proportional error, suggesting good agreement across the range of measurements.

**Figure 3:**
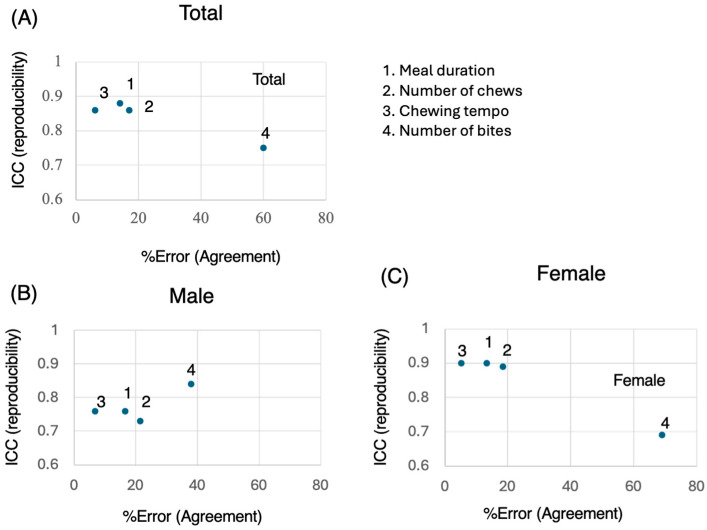
Reproducibility and agreement across measures. The X- and Y-axes represent the percent error (agreement) and intraclass correlation coefficient (ICC) (reproducibility), respectively, for each measured variable. (**A**) Total; (**B**) Male; (**C**) Female. Higher ICC values indicate better reproducibility, whereas lower percent error values suggest stronger agreement between repeated measures.

The median difference in meal duration between the two measurements was −6.0 s (95% CI: −46.0–25.0) for the total group, with LOAs ranging from −350.2–71.2 s (Table 4, Figure 2A and Figure 3). The percentage of participants with values within the LOA was 93.9%, with a percentage error of 14%. The median Bland–Altman ratio was −0.012 (95% CI: −0.068–0.036), and the ratio-based LOA ranged from −0.38–0.13. The Wilcoxon signed-rank test revealed no significant difference (*p* = 0.16). The ICC was 0.88, and the Spearman’s rho was 0.88 (Table 4, Figure 2A and Figure 3).

In the analysis stratified by sex, males presented greater variability (median difference: −19.0 s, 95% CI: −117.0–31.0 s; LOA: −292.9–44.8 s; percentage error: 21.4%) than females did (median difference: 19.5 s, 95% CI: −56.0–31.0 s; LOA: −340.8–105.3 s; percentage error: 13.4%). The ICCs were 0.73 and 0.90 for males and females, respectively (Table 4, Figure 2A and Figure 3).

#### 3.3.2. Number of Chews

The median difference in the total number of chews was −16.0 (95% CI: −73.0–22.0), with an LOA ranging from −492.0–349.8. The ratio-based analysis revealed a median of −0.024 (95% CI: −0.056–0.030), with an LOA ranging from −0.40–0.27 (Table 4, Figure 2B and Figure 3). The ICC was 0.86, and the Spearman’s rho was 0.88. No significant difference was detected via the Wilcoxon test (*p* = 0.30) (Table 4, Figure 2B).

In terms of sex, males presented greater negative bias (−30.0, 95% CI: −115.0–30.0) and a narrower LOA (−412.2–95.0), whereas females presented near-zero bias (1.5, 95% CI: −74.0–80.0). The ICC was greater in females (0.89) than in males (0.76) (Table 5, Figure 2B and Figure 3).

#### 3.3.3. Chewing Tempo

The chewing tempo showed high reproducibility, with a median difference of −0.38 (95% CI: −1.88–1.49), an LOA of −10.18–14.1, and a median ratio of −0.005 (95% CI: −0.022–0.012) (Table 4, Figure 2C and Figure 3). The ICC was 0.86, and the Spearman’s rho was 0.84. No significant difference was found (*p* = 0.805) (Table 4).

Males had slightly lower reproducibility (ICC: 0.76, percentage error: 6.8%) than females did (ICC: 0.90, percentage error: 5.3%) (Table 5, Figure 2C and Figure 3).

#### 3.3.4. Number of Bites

The median difference in the number of bites was −2.0 (95% CI: −9.0–1.0), with an LOA ranging from −37.4–9.8 and a median ratio of −0.11 (95% CI: −0.35–0.069) (Table 4, Figure 2D). The percentage error was relatively high (60%) (Table 4), and the Wilcoxon test revealed a significant difference (*p* = 0.034) (Table 5).

Among females, the median difference was −9.0 (95% CI: −13.0–−2.0), with a significantly narrower LOA (−41.9–7.7), and the Bland–Altman ratio also indicated systematic bias (median: −0.32, 95% CI: −0.45–−0.093, *p* = 0.006) (Table 4, Figure 2D). The ICC was 0.69 for females and 0.84 for males (Table 5).

### 3.4. ICCs vs. %Error

Finally, we present a figure showing the % error (agreement) on the horizontal axis and the ICC (reproductivity) on the vertical axis (Figure 3). The duration, number of chews, and chewing tempo were within 20% for both males and females (Figure 3). On the other hand, the % error for the number of bites tended to be greater for females (Figure 3). In terms of sex, reproducibility tended to be greater in the female group than in the male group. With respect to the statistical significance of the Wilcoxon test, there were no meaningful significant differences in the meal duration measurements between the two time points (Figure 3). Thus, the agreement and reproductivity of the meal duration, number of chews, and chewing tempo in this food test were high. The number of bites may vary significantly due to subjective differences and individual eating styles, leading to lower reproducibility.

## 4. Discussion

In this study, the reproducibility and agreement of meal duration, the number of chews, the chewing tempo, and the number of bites were examined by having participants eat the same meal (salmon bento) at a 2-week interval. The reproducibility and agreement of meal duration, the number of chews, and the chewing tempo were high; the reproducibility (ICC) of the number of bites was high, but the agreement was low. The median difference was greater for the second meal than for the first meal in females. The Wilcoxon_*p* test value was also significant at *p* = 0.0063. This trend was different from that of males. The meal duration, number of chews, and chewing tempo were the same even if they were measured at different times for the same meal, but the number of bites could change with each meal, and this tendency was more pronounced for females. Therefore, the meal duration, number of chews, and chewing tempo of test meals are highly reproducible and consistent with indices that define an individual’s eating behavior.

In the present study, the meal duration and number of bites were significantly greater in the female group than in the male group, and the number of chews, although not statistically significant, tended to be greater in the female group. The same results were observed in our previous studies using pizza and hamburger lunches [11,12]. Similarly, in a study in which another group was fed 152 g of steamed rice, males had a faster eating rate (*p* < 0.05) than females did, and females habitually chewed more (*p* < 0.05) and had a longer meal duration (*p* < 0.01) than males did (Soojin Park et al.) [17]. In addition, in the present study, meal duration was also correlated with sex, in addition to the number of chews, the number of bites, and age. Possible reasons why women tend to spend more of their time eating and chewing more frequently include physiological factors, psychological and behavioral factors, and sociocultural factors. First, women may need more chewing to process the same food because of lower chewing strength [30]. Estrogen also has an appetite-suppressing effect, which may lead to more mindful eating behavior [31,32]. Women are often more concerned with weight control and body image, which leads to intentional slow eating to avoid overeating. This behavior is known as “restrained eating” [33,34]. In Japan, women may engage more in conversation or caregiving roles during meals (e.g., serving others first), leading to longer mealtimes. Women are often expected to eat politely, avoid making noise while chewing, and not rush, resulting in more chewing and slower eating. Therefore, it is necessary to stratify by sex when examining eating behaviors such as meal duration, the number of chews, and the number of bites. Moreover, the associations between meal duration and the number of chews or bites are independent of the type of meal.

The present dietary study revealed that the characteristics of individual eating behavior (meal duration, number of chews, and chewing tempo) are reproducible. Unfortunately, only a few studies to date have examined the reproducibility of measurements of meal duration and the number of chews by having participants eat the same meal repeatedly. Many previous studies have examined the reproducibility of the food frequency questionnaire (FFQ), and studies of questionnaires that are similar to the FFQ have examined the reproducibility of dietary intake [35,36,37,38]. When daily meals are considered, considerable fluctuations may occur, but over longer periods, such as seven days or one month, the average dietary intake of healthy individuals does not change. One study assessed food intake reproducibility via two repeated ad libitum meals separated by one week [13]. ICCs and Bland–Altman analysis (median difference ± limits of agreement) were used to evaluate repeatability, following approaches established for ad libitum energy intake and thermogenic response measurements. The correlation between ad libitum energy intake on the two test days was r = 0.654 (R2 = 0.428, *p* = 0.001) [13]. Thus, the ad libitum test meal used to measure spontaneous energy intake is reproducible. As meal duration is correlated with energy intake [39], these data suggest that measurements of meal duration and the number of chews during dietary testing are generally reproducible.

Compared with the other test items, the number of bites was particularly low in women. For the possibility of uneven serving of the meals, both the rice and salmon bento are measured and provided in the factory, so it is unlikely that there would be any variation in quantity. If such variation were to occur, similar inconsistencies would be expected in terms of meal duration and the number of chews. If the issue were due to the measurement method, the same results would probably be observed in men. We focused on the fact that proportional errors occurred only in women. In other words, people with a greater number of bites per mouthful tend to eat with even more bites. Perhaps, in the case of women, during the second session, they unconsciously reduced their bite count by trying to eat more slowly. This may suggest that the difference in eating behavior by sex is due to psychological factors [33,34].

One limitation of this study is the lack of an a priori power analysis due to its exploratory design. Because the sample size is small, it is necessary to consider issues such as reduced statistical power, instability of estimates, wider confidence intervals, and a greater likelihood that the sample does not represent the population, making it difficult to apply the results to other groups.

Second, for the number of bites, the reproducibility was high, but the agreement was low. In our study, the number of bites was taken to indicate the number of times a person took a break from chewing during meals. Unlike pizza or hamburger bento (hamburger steak and broccoli), salmon bento contains six types of side dishes. Therefore, since salmon bento requires the use of chopsticks more than hamburger bento does, the way in which chopsticks are used could have a greater and more unstable effect on the number of bites. In the measurements for salmon bento, proportional error was observed among women, suggesting that foods with fewer bites are less likely to produce errors. Furthermore, with even more complex multi-course meals, reproducibility and consistency are thought to decrease. Therefore, it may be better to use relatively uniform food items, such as pizza or rice only, to evaluate the number of bites.

Additionally, while the psychological state at the time was not evaluated in this test, such factors may still influence meal duration and the number of chews. The measurement location differed from that of an ordinary dining area, being an environment without visible surroundings or noise and lacking the lively atmosphere typical of a cafeteria. Despite not being able to rule out psychological effects, the reproducibility of the results was surprising. Currently, we plan to examine whether there is a real correlation between the time required for meals in daily life and the time spent eating during the test meals. Therefore, it is necessary to conduct studies that bridge the gap between eating behavior metrics measured by such test meals and real-life situations.

Finally, because of the small number of participants in this study, analysis by age group was not possible. However, since an effect of age on meal duration was observed, further analysis by age will be necessary as the number of participants increases.

## 5. Conclusions

In conclusion, the reproducibility and agreement of meal duration, number of chews, and chewing tempo using the test meal (salmon bento lunch box and steamed rice) were high. On the other hand, the agreement of the number of bites was low, especially in females. In future studies, we are planning to examine whether there is a correlation between meal duration and the number of chews during the test meal and between meal duration and energy intake for each meal as well as the seven-day dietary record. Owing to the high reproducibility and concordance of this test meal (salmon bento lunch box), we believe that meal duration, the number of chews, and the chewing tempo measured via at least one test meal (rice and salmon bento lunch box) can be used as parameters to describe eating behaviors that are specific to individuals.

## Figures and Tables

**Table 1:** Characteristics of the participants in this study.

	Total (*n* = 33)	Male (*n* = 15)	Female (*n* = 18)
Age (yo)	41.6 (8.4)	42.0(8.2)	41.2(8.9)
BMI (kg/m^2^)	22.5 (3.0)	23.7(2.9)	21.5 (2.7)
eGFR	83.1 (13.1)	82.4 (11.9)	83.8 (14.3)
HbA1c (%)	5.4 (0.3)	5.5(0.3)	5.4(0.3)
UA (mg/dL)	4.8 (1.4)	5.7 (1.1)	4.0 (1.1)
TC (mg/dL)	203.5 (31.1)	204.9 (34.1)	202.3 (29.3)
TG (mg/dL)	103.8 (71.5)	133.6 (82.0)	78.9 (51.5)
HDL-C (mg/dL)	64.3 (14.1)	56.0 (14.1)	71.2 (9.8)
Energy intake (kcal)	1568.6 (432.0)	1717.6 (451.0)	1444.4 (384.5)
Protein intake (g)	59.0 (17.0)	61.4 (18.2)	56.9 (16.1)
Fat intake (g)	50.7 (13.3)	53.0 (15.1)	48.8 (11.8)
Carbohydrate intake (g)	200.6 (65.3)	226.8 (75.1)	178.8 (47.7)
Dietary fiber intake (g)	9.4 (3.4)	9.5 (2.8)	9.4(4.0)
Salt intake (g)	9.1 (2.4)	10.0 (2.5)	8.2 (1.9)

The data are presented as the means (SDs). Abbreviations: BMI, body mass index; eGFR, estimated glomerular filtration rate; UA, uric acid; TC, total cholesterol; TG, triglyceride; HDL-C, high-density lipoprotein-C.

**Table 2:** First and second meal durations, number of chews, eating tempo, and number of bites.

	Total (*n* = 33)	Male (*n* = 15)	Female (*n* = 18)	*p*
Meal duration_1st	654.0 (229.0)	560.4 (128.7)	731.9 (266.3)	0.023
Meal duration_2nd	696.3 (275.0)	608.3 (180.5)	769.7 (320.9)	
Number of chews_1st	854.7 (319.3)	752.5 (203.3)	938.1 (375.9)	0.083
Number of chews_2nd	880.4 (329.1)	818.1 (255.9)	932.3 (379.0)	
Eating tempo_1st	82.6 (11.0)	84.1 (12.2)	81.2 (12.2)	0.46
Eating tempo_2nd	81.7 (12.1)	84.9 (10.2)	79.0 (13.1)	
Number of bites_1st	22.2 (12.8)	17.1 (9.9)	26.4 (13.7)	0.036 *
Number of bites_2nd	27.2 (22.3)	16.5 (12.5)	36.1 (25.0)	

We measured the duration, number of chews, tempo, and number of bites of a test meal (salmon bento lunch box and steamed rice) among 33 participants aged 20–60 years (mean 41.6) who ate twice at 2-week intervals. A *t* test and * Mann-Whitney U test were conducted. The data are presented as the means (SDs). Red font indicates statistical significance (e.g., *p* < 0.05). Descriptive statistics for both the first and second measurements were reported to show potential practice effects or variation across repeated assessments. However, to maintain the statistical independence of the observations, only the first measurement was used for sex comparisons.

**Table 3:** Multivariate regression analysis of meal duration and several factors (number of chews, chewing tempo, and number of bites).

	Model 1		Model 2		Model 3		Model 4	
	B [95% CI]	*p*	B [95% CI]	*p*	B [95% CI]	*p*	B [95% CI]	*p*
Number of Chews	0.64 [0.53, 0.74]	<0.001						
Chewing Tempo			3.8 [–3.3, 10.9]	0.28				
Number of Bites					10.4 [5.1, 15.8]	<0.001		
Age							−9.2 [−17.9, −0.5]	0.04
Sex (Male: 0, Female: 1)	53.8 [–12.5, 120.0]	0.11	182.6 [27.9, 337.2]	0.02	74.5 [–61.6, 210.6]	0.27	164.4 [18.8, 310.0]	0.03

Red font indicates statistical significance (e.g., *p* < 0.05).

**Table 4:** Summary of agreement by Bland–Altman analysis.

Group_Metric	Median_Diff_CI	LOA_Lower	LOA_Upper	Percent Within_LOA	%_Error	BA_Median_Ratio_CI
Total_meal duration (s)	−6.0 [−46.00, 25.00]	−350.2	71.2	93.9	14	−0.012 [−0.068, 0.036]
Male_meal duration (s)	−19.00 [−117.00, 31.00]	−293	44.8	86.7	21.4	−0.028 [−0.18, 0.057]
Female_meal duration (s)	19.50 [−56.00, 31.00]	−340.8	105.3	88.9	13.4	0.025 [−0.078, 0.049]
Total_number of chews (times)	−16.00 [−73.00, 22.00]	−492	349.8	93.9	16.9	−0.024[−0.056, 0.030]
Male_number of chews (times)	−30.00 [−115.00, 30.00]	−412.2	95	86.7	16.5	−0.042 [−0.15, 0.030]
Female_number of chews (times)	1.5 [−74.00, 80.00]	−339.5	364.4	88.9	18.5	0.004 [−0.056, 0.070]
Total_chewing tempo (cpm)	−0.38 [−1.88, 1.49]	−10.2	14.1	93.9	6	−0.005 [−0.022, 0.012]
Male_tempo (cpm)	−2.23 [−4.49, 2.24]	−12.7	12.8	86.7	6.8	−0.023 [−0.048, 0.020]
Female_tempo (cpm)	0.6 [−0.66, 2.45]	−3.7	13.3	88.9	5.3	0.006 [−0.009, 0.038]
Total_number of bites (times)	−2.00 [−9.00, 1.00]	−37.4	9.8	93.9	60	−0.11 [−0.35, 0.069]
Male_number of bites (times)	1.00 [−2.00, 3.00]	−11.6	11.3	86.7	37.9	0.069 [−0.11, 0.40]
Female_number of bites (times)	−9.00 [−13.00, −2.00]	−41.9	7.7	88.9	68.9	−0.32 [−0.45, −0.093]

We measured the duration, number of chews, chewing tempo, and number of bites of a test meal (salmon bento lunch box and steamed rice) among 33 participants aged 20–60 years (mean 41.6) who ate twice at 2-week intervals. Abbreviations: LOA, limits of agreement: BA, Bland-Altman: CI, confidence interval.

**Table 5:** Summary of reproducibility via the Wilcoxon signed-rank test, the ICC, and Spearman_rho.

Group_Metric	Wilcoxon_p	ICC	Spearman_rho
Total_meal duration (s)	0.16	0.88	0.88
Male_meal duration (s)	0.24	0.73	0.88
Female_meal duration (s)	0.39	0.9	0.88
Total_number of chews (times)	0.3	0.86	0.88
Male_number of chews (times)	0.11	0.76	0.83
Female_number of chews (times)	0.97	0.89	0.91
Total chewing tempo (cpm)	0.81	0.86	0.84
Male chewing tempo (cpm)	0.45	0.76	0.76
Female chewing tempo (cpm)	0.25	0.9	0.86
Total_number of bites (times)	0.034	0.75	0.83
Male_number of bites (times)	0.62	0.84	0.69
Female_number of bites (times)	0.0063	0.69	0.81

We measured the duration, number of chews, chewing tempo, and number of bites of a test meal (salmon bento lunch box and steamed rice) among 33 participants aged 20–60 years (mean 41.6) who ate twice at 2-week intervals. Abbreviations: ICC, intraclass correlation coefficient; Wilcoxon, Wilcoxon signed-rank test.

## Data Availability

Some or all datasets generated during and/or analyzed during the current study are not publicly available but are available from the corresponding author upon reasonable request.

## Abbreviations

BDHQ	Brief-Type Self-Administered Diet History Questionnaire
